# The Crosstalk between Gut Microbiota and White Adipose Tissue Mitochondria in Obesity

**DOI:** 10.3390/nu15071723

**Published:** 2023-03-31

**Authors:** Luca Colangeli, David Israel Escobar Marcillo, Valeria Simonelli, Egidio Iorio, Tommaso Rinaldi, Paolo Sbraccia, Paola Fortini, Valeria Guglielmi

**Affiliations:** 1 Department of Systems Medicine, University of Rome Tor Vergata, 00133 Rome, Italy; 2 Obesity Medical Center, University Hospital Policlinico Tor Vergata, 00133 Rome, Italy; 3 Section of Mechanisms, Biomarkers and Models, Department of Environment and Health, Istituto Superiore di Sanità, Viale Regina Elena, 299, 00161 Rome, Italy; 4 High Resolution NMR Unit, Core Facilities, Istituto Superiore di Sanità, Viale Regina Elena, 299, 00161 Rome, Italy

**Keywords:** obesity, gut microbiota, mitochondria, white adipose tissue, crosstalk

## Abstract

Adipose tissue (AT) dysregulation is a key process in the pathophysiology of obesity and its cardiometabolic complications, but even if a growing body of evidence has been collected over recent decades, the underlying molecular basis of adiposopathy remains to be fully understood. In this context, mitochondria, the intracellular organelles that orchestrate energy production and undergo highly dynamic adaptive changes in response to changing environments, have emerged as crucial regulators of both white (WAT) and brown adipose tissue (BAT) metabolism and function. Given that the gut microbiota and its metabolites are able to regulate host metabolism, adipogenesis, WAT inflammation, and thermogenesis, we hypothesize that their frequently observed dysregulation in obesity could affect AT metabolism by exerting direct and indirect effects on AT mitochondria. By collecting and revising the current evidence on the connections between gut microbiota and AT mitochondria in obesity, we gained insights into the molecular biology of their hitherto largely unexplored crosstalk, tracing how gut microbiota may regulate AT mitochondrial function.

## 1. Introduction

Obesity is one of the most important challenges of this century. According to World Health Organization (WHO) data, in 2016 about 13% of the world’s adult population were obese. The prevalence of obesity has dramatically increased in the last 50 years, reaching epidemic proportions and becoming an important health issue, even in developing countries that in the past mainly faced malnutrition [1]. Projections report that obesity will affect more than 30% of adults in European countries in less than 15 years, and in the United States, this threshold has already been largely surpassed [2].

A combination of genetic, behavioral, and environmental variables produces a chronic positive energy balance, and energy in excess is stored as lipid droplets in adipose tissue (AT) [3]. The progressive increase in fat deposits determines the expansion of AT, but when this process overcomes the angiogenesis and oxygenation of the enlarged adipocytes, an inflammatory state of altered cytokine secretion and macrophage infiltration begins [4]. Insulin resistance is the main consequence of this chronic low-grade inflammation and is responsible for an increased risk of cardiometabolic and also oncological diseases [5]. AT dysregulation is therefore the key process in the pathophysiology of obesity, which has been correctly defined an “adiposity-based chronic disease” by the European Association for the Study of Obesity [6].

Even if an enormous amount of evidence regarding obesity has been collected in recent years, we still need to understand the biological mechanisms underlying this disease. The scientific community agrees that obesity is closely related to imbalanced cellular energy metabolism, in particular to mitochondrial metabolism [7], but the role of gut microbiota is also gaining greater interest. Gut microbes can modulate the host’s appetite, intestinal permeability, energy absorption, and overall lipid and glucose metabolism [8]. Alterations in gut microbiota have been questioned as being involved in the development of metabolic diseases, such as insulin resistance and type 2 diabetes (T2D), that typically affect individuals with obesity [9]. Given that gut microbiota and microbial metabolites have increasingly been recognized to influence AT metabolism, we hypothesize that the crosstalk between gut microbiota and AT could be mediated by the adipocytes’ mitochondria.

Thus, the aim of this review is to summarize the current evidence on the connections between gut microbiota and AT mitochondria in obesity.

## 2. White and Brown Adipose Tissue

Historically, AT has been considered as an inert connective tissue with only cushioning and thermal insulation functions, but in recent years it has acquired the status of an endocrine organ because of its important effects on the regulation of metabolism through the secretion of hormones (such as leptin and adiponectin) and cytokines (also called “adipokines”) [10].

Adipocytes are those cells primarily characterizing AT, which nonetheless, is also composed of many other cell types, such as pre-adipocytes, fibroblasts, vascular endothelial cells, and immune cells [11].

**Figure 1:**
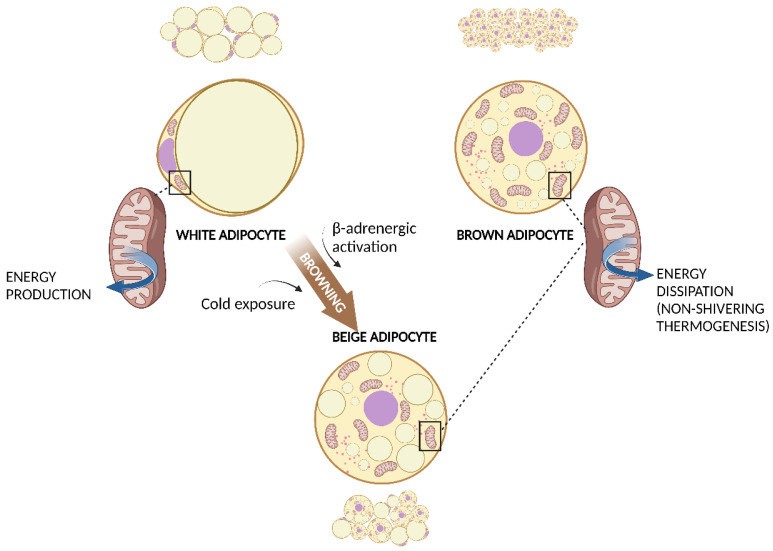
WAT, BAT, and WAT browning. White adipocyte has one large droplet in the centre of the cell that compresses nucleus and mitochondria at one pole. Brown adipocyte has multiple small lipid droplets and more mitochondria, spread out between the droplets. Beige adipocyte has intermediate characteristics. Cold exposure and β-adrenergic activation determine the browning of WAT. Both brown and beige mitochondria are involved in non-shivering thermogenesis. Created with BioRender.com (accessed on 13 March 2023). Abbreviations: BAT, brown adipose tissue; WAT, white adipose tissue.

Altogether, we can distinguish two main types of adipose tissue that differ in cell structure, histology, amount, anatomic location, and function: white adipose tissue (WAT) and brown adipose tissue (BAT) (Figure 1).

WAT is composed of white adipocytes: large cells with a diameter of 20–150 μm, almost completely occupied by a single, large lipid droplet that pushes the nucleus and other organelles to one pole of the cell. Brown adipocytes are smaller cells with a diameter of about 10–25 μm and many lipid droplets that give the cell a multilocular aspect. Another important characteristic of brown adipocytes is the higher number of mitochondria, which determines the typical brown coloration of BAT under microscopy [12].

WAT is more abundant than BAT in humans. There are two main anatomic districts where WAT can be found and where it assumes different functions: the subcutaneous district (subcutaneous adipose tissue, SAT) and the visceral district (visceral adipose tissue, VAT) [13]. SAT is localized just beneath the skin and represents about the 85% of total body fat in a normal weight adult. It can be found principally in abdominal, gluteal, and femoral depots, with differences influenced also by the gender (in women, it is more abundant in the gluteal–femoral region) [14]. Other than having a mechanic protective function acting as a cushion against external traumatic and thermal stress, SAT plays an important role in glucose and lipid homeostasis [15]. VAT is composed of the fat deposits in the abdominal cavity: omental (hanging from the stomach), mesenteric (along the intestine) and epiploic (near the colon) fat. Typically accounting for less than 20% of the total body fat, VAT expansion is associated with the development of the metabolic complications of obesity and a substantial increase in cardiovascular risk [16]. Waist circumference can be considered to be a valid estimator of VAT accumulation and denotes a more robust independent risk factor for insulin resistance, T2D, dyslipidemia, and atherosclerosis than body mass index (BMI) [17,18].

In addition to SAT and VAT, there are ectopic deposits of fat that infiltrate other anatomic structures such as skeletal muscle, arteries, and myocardium, which despite their smaller amounts play a significant role in the development of obesity complications. Indeed, intermuscular fat has been linked to insulin resistance and muscle catabolism, whereas the epicardial AT surrounding the myocardium and coronary arteries plays a role in the pathogenesis of coronary atherosclerosis and atrial arrhythmias [19,20].

Unlike other mammals such as small rodents, in humans BAT represents a minimum percentage of body weight. BAT volume can be assessed with 18 fluoro-deoxy-glucose positron emission tomography/computed tomography (18FDG-PET/CT) [21]. There is a higher amount in infants (when skeletal muscles have not developed enough to maintain body temperature after cold exposure through shivering thermogenesis) and the amount tends to decrease thereafter in adulthood, remaining in the supraclavicular region and in small quantities around the great vessels in the paravertebral and mediastinal areas [22].

The varying distribution and differences in morphology and histology between WAT and BAT reflect their different functions. WAT plays an important role in energy homeostasis, as it is able to store surplus energy in the form of triglycerides that can be then mobilized during periods of high energy demand [23]. In presence of a chronically positive energy balance, WAT can expand following two main pathways: adipocyte hyperplasia (increase in the number of adipocytes thanks to the proliferation and differentiation of mesenchymal stem cells) and hypertrophy (enlargement of existing adipocytes due to increased lipid storage). Although hyperplasia has been associated with a more metabolically healthy condition, adipocyte hypertrophy is accompanied by impaired AT angiogenesis, fibrosis, oxidative stress, and inflammatory cell proliferation that lead to low-grade chronic inflammation and insulin resistance [24], two well-known complications of obesity that contribute to increasing cardiovascular risk [25].

WAT also exerts an endocrine function, as it produces enzymes involved in steroid hormone metabolism (e.g., aromatase) and secretes adipokines which control energy, lipid, and carbohydrate metabolism and which can modulate immune system activity not only locally, but at the systemic level [26]. Leptin is a peptide hormone synthesized by adipose cells in response to food intake and provides the hypothalamus with information to control feeding and regulate body weight homeostasis. Leptin circulation levels are proportional to body fat mass and reflect the body’s energy reserves. Increased leptin levels induce anorexigenic factors (such as cocaine–amphetamine-related transcript) and suppress orexigenic neuropeptides (such as neuropeptide Y), thereby reducing food intake [27]. On the contrary, adiponectin is secreted in higher quantities when there is a reduction in body fat, reflecting a chronically negative energy balance, and is associated with anti-inflammatory and insulin-sensitizing effects characteristic of weight loss [28].

As well as WAT, BAT is also involved in energy homeostasis, but its role is diametrically opposite: expending energy instead of storing it. BAT is the site of the so-called non-shivering thermogenesis. Different from shivering thermogenesis produced by the involuntary contraction of muscles caused by cold exposure, non-shivering thermogenesis is carried out by BAT mitochondria via the uncoupling protein-1 (UCP-1) [29]. Brown adipocytes present a large number of small lipid droplets which are more easily accessible for hydrolysis and oxidation of free fatty acids (FFAs). UCP-1, expressed within the inner membrane of mitochondria, uncouples oxidation from subsequent adenosine diphosphate phosphorylation and thereby energy is dissipated as heat. Cold exposure is the principal activator of BAT and the effect of cold is mediated via the activated sympathetic nervous system. Interestingly, chronic cold exposure causes an increase in UCP-1 expression and mitochondrial content in white adipocytes in a process known as browning (Figure 1). These adipocytes have characteristics that are in between those of white and brown adipocytes and are called “brite” or beige adipocytes, and they can produce heat with UCP-1, similarly to BAT [30]. Chronic exercise has also been associated with subcutaneous WAT browning [31]. As BAT activation has been associated with a healthier inflammatory profile and a reduction in insulin-resistance, WAT browning is considered an attractive strategy for the prevention of metabolic diseases [32].

## 3. Mitochondria in WAT and BAT

Mitochondria are cytoplasmic organelles that play a critical role in the energy metabolism of all eukaryotic cells and generate energy in the form of adenosine triphosphate (ATP). There are two principal ways of producing ATP in mitochondria: (1) oxidation of carbohydrates, fats, and proteins through the tricarboxylic acid (TCA) cycle and (2) β-oxidation of fatty acids [33]. The mitochondria play a central role in all cellular processes by catalyzing the oxidation of fuel molecules (glucose, fat acid, and amino acids) and transforming the electrons to molecular oxygen with concomitant energy transduction into ATP (oxidative phosphorylation, and OXPHOS) [34]. The OXPHOS includes five enzymatic complexes and two mobile electron carriers (NADH and FADH2) that work in a mitochondrial respiratory chain (I: NADH–coenzyme Q reductase; II: succinate–coenzyme Q reductase; III: coenzyme QH2 cytochrome-c reductase; IV: cytochrome-c oxidase; V: ATP synthase) [35].

Mitochondria are surrounded by a double-membrane system of inner and outer membranes. The inner membrane is folded in cristae that increase the overall surface where OXPHOS takes place under aerobic conditions [33]. A unique characteristic of mitochondria is the presence of a proper genome, a circular mitochondrial DNA (mtDNA) that mainly encodes for proteins related to OXPHOS which are added to over one thousand other mitochondrial proteins encoded by nuclear DNA (nDNA) [36]. Mitochondria are very dynamic organelles capable of meeting bioenergy or oxidative challenges by changing their morphology and protein content. Through fusion, mitochondria can exchange proteins and mtDNA to improve their function, then they can divide again by fission, producing newly restored mitochondria [37]. Aged and damaged mitochondria which can no longer be restored are selectively degraded by lysosomes in a process called mitophagy [38]. Impairment to these processes and overall mitochondrial homeostasis can lead to the development of metabolic diseases.

In WAT, adipocytes contain a relatively low mitochondrial mass per overall cell size compared with other cell types [39], but mitochondria exert a fundamental role in regulating lipid turnover. In presence of a positive energy balance, mitochondria generate the intermediary metabolites needed for lipogenesis, such as acetyl-CoA for fatty acid synthesis and esterification with glycerol-3-phosphate into triglycerides. Then, during fasting or in conditions of high energy demand, adipocytes release FFAs via lipolysis, thanks to hormone-sensitive lipase and adipose triglyceride lipase [40].

Mitochondria are also involved in adipocyte differentiation and adipogenesis [41]. During adipogenesis, mitochondrial oxygen consumption and the amount of mitochondrial proteins increase [42]. Mitochondrial biogenesis shares many key regulators with adipogenesis, such as the peroxisome proliferator-activated receptor gamma (PPARγ) and the peroxisome proliferator-activated receptor gamma coactivator-1alpha (PGC-1α), with which it co-activates [43]. Activation of PPARγ nuclear receptors prompts the transcription of genes related to a wide spectrum of biological functions, from mitochondrial and turnover to antioxidant defense and immune response. Rosiglitazone, a PPARγ agonist originally developed for the treatment of T2D, promotes mitochondrial biogenesis and remodeling in WAT that improve the entire body’s energy metabolism and insulin sensitivity [44]. PGC-1α is a key component of mitochondrial biogenesis and induced by reactive oxygen species (ROS) which also regulate adipogenesis, as ROS are required for the differentiation of preadipocytes to adipocytes. Imbalance in ROS levels triggers mitochondrial and adipocyte dysfunction resulting in WAT oxidative stress and inflammation [45].

Moreover, mitochondria may also be involved in WAT adipokine secretion. Adiponectin and mitochondrial biogenesis are interconnected, as one stimulates the other. It has been demonstrated that adiponectin inhibits mitochondrial mediated apoptosis and, in turn, mitochondrial biogenesis positively regulates adiponectin secretion, in a virtuous circle which improves glucose metabolism and insulin sensitivity [46]. On the contrary, impaired mitochondrial function in WAT may explain the reduced adiponectin levels observed in obesity associated with low-grade chronic inflammation and cardiovascular diseases [47]. With regard to leptin, it has been reported that mitochondrial function, rather than mitochondrial biogenesis or mass, can be improved by administering leptin [48]; however, further studies are required to determine whether there is a direct relationship with AT mitochondria.

Most of our knowledge about the metabolism and function of mitochondria in WAT comes from studies on SAT. Less is known about VAT mitochondria, particularly in conditions of good health. In rats, VAT presents higher mitochondrial density, mtDNA content, mitochondrial enzymes, and respiration as compared to SAT [49]. In obesity, mitochondrial respiration and phosphorylation activity seem significantly lower in VAT, supporting the hypothesis of a dysfunctional activity of VAT mitochondria [50].

We previously reported differences in WAT and BAT morphology, histology, distribution, and function, but BAT also shows specific mitochondrial characteristics (Table 1). First of all, BAT appears brownish in microscopic images just because of the higher expression of cytochrome oxidase, an iron-containing heme cofactor, that indicates higher mitochondrial concentrations [41]. Transmission electron microscopy images from rat interscapular BAT show that mitochondria in BAT are bigger, have a spherical shape, and contain more packed cristae [51]. Their distinctive feature, both in rodents and in humans, is the higher expression of UCP-1 [52] which reflects their main function of heat generation through non-shivering thermogenesis [53].

In brown adipocytes that are highly vascularized and innervated cells, mitochondria are immersed amongst numerous small lipid droplets [54]. Cold induces sympathetic stimulation of brown adipocytes via β3-adrenergic receptors in rodents but predominantly by β1-adrenergic receptors in humans [55]. The β-adrenergic receptor couples with a Gs protein which activates adenylyl cyclase, leading to the formation of cAMP as a secondary messenger. Subsequently, cAMP promotes the activation of cytosolic lipolytic enzymes (hormone-sensitive lipase, adipose triglyceride lipase, and perilipin) and lipolysis produces FFAs [56]. Through the carnitine cycle, FFAs are converted into long chain acyl-carnitine esters that are imported into the mitochondrial matrix and then converted back into acylCoA, serving as a substrate for the TCA cycle [57]. Meanwhile, FFAs directly activate UCP-1, which dissipates the electrochemical proton gradient produced by the respiratory chain, thus catalysing the leak of protons across the mitochondrial inner membrane. In this way, energy from the oxidation of respiratory substrates that would otherwise be utilized for ATP synthesis is released as heat [58].

## 4. Obesity and Mitochondrial Metabolism in WAT and BAT

Lipid and glucose metabolism and insulin sensitivity are strongly affected by obesity and overweight conditions [59].

**Figure 2:**
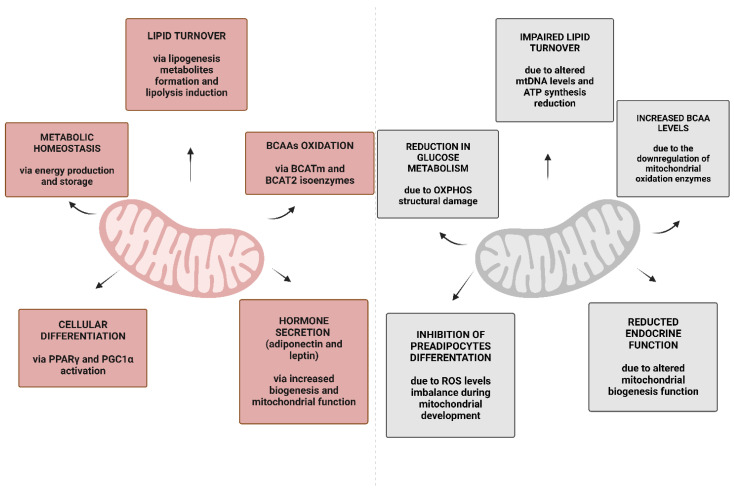
Physiological functions of WAT mitochondria and common impairments in obesity. The left panel (in pink) refers to the physiological condition where some of the main functions of WAT mitochondria are presented. In the right panel (in grey), the same functions are impaired in obesity. Created with BioRender.com (accessed on 13 March 2023). Abbreviations: BCAAs, branched-chain amino acids; BCAT, branche- chain amino acid aminotransferase; OXPHOS, oxidative phosphorylation; PGC-1α, peroxisome proliferator-activated receptor gamma coactivator-1α; PPARγ, peroxisome proliferator activated receptor-γ; ROS, reactive oxygen species.

There is growing evidence that indicates that mitochondria play a central role in WAT metabolic homeostasis [40,60,61]. Mitochondria are biochemical powerhouses which not only provide energy to the cell by coupling the TCA cycle with OXPHOS, but are biochemical hubs, where a complex network of reactions catabolize different substrates and generate metabolites involved in all the anabolic and catabolic processes of the cell (Figure 2).

In recent years, altered mitochondrial homeostasis has emerged as a possible cause of obesity-related metabolic impairments. Some of the most interesting findings in this field come from studies on twins discordant for BMI (lean versus obese), that allow distinguishing of acquired features from those that are genetically caused. In particular, Heinonen et al. reported lower expression of genes encoding both mitochondrial proteins and PGC-1α, lower mtDNA copy number, and reduced levels of OXPHOS complex subunits in obese compared to lean co-twins [40]. The expression of PGC-1α and activities of the complexes I to IV were downregulated in the SAT of patients with obesity as compared with controls [62]. Moreover, the downregulation of nuclear transcription factors (such as PGC-1α) results in downregulation of the adipogenesis. The reduced capacity of pre-adipocytes to differentiate into adipocytes determines, in a chronic positive energy balance, the enlargement of AT through hypertrophy more than through hyperplasia, a processwell-known to be associated with inflammation and insulin-resistance [63]. Effectively, it has been found that in the VAT of metabolically healthy obese patients (that is, obese patients who do not present the characteristics of metabolic syndrome) adipocytes are smaller than in obese patients with metabolic complications [64]. Several preclinical and human studies suggest there is a reduction in oxidative metabolism AT mitochondria in obesity [39,44].

Recently it has been reported that accumulation of succinate, an intermediary in TCA cycle (through activation of UCP1), plays a role in activating AT thermogenesis [65,66] suggesting a novel role for this mitochondrial metabolite in AT differentiation.

From a metabolic point of view, the mitochondria of AT are very flexible and adaptable to external stimuli. The exposure to nicotinamide riboside (NR) of human white adipocytes can shift differentiation to beige adipocytes [67]. Under experimental conditions, NR did not induce poly-ADP-ribosepolymerase (PARP) activity but enhanced sirtuin 1 (SIRT1) activation and reactive species, which play a key role in adipocyte browning [67]. Furthermore, it has been demonstrated that a high-fat sucrose diet enhanced glyceroneogenesis and shifted BAT metabolism toward the WAT phenotype with triacylglycerol (TAG) synthesis and enhanced fatty acid esterification, favoring the development of obesity [68].

AT is the major site where carbohydrates are converted to fat [69,70]. In particular, during excess of nutrients (high energy status), malonyl-CoA, produced by both glucose and lipid metabolism, inhibits fatty acids from being imported to mitochondria through the CPT1 transporter, thus decreasing fatty acid oxidation in the mitochondria [71]. On the contrary, in a state of low energy, the activation of AMPK leads to enhanced CPT1 activity and β-oxidation in mitochondria. Mitochondrial fatty acid oxidation is impaired in obesity and SAT [39].

AT is one of the main tissue types with higher mitochondrial catabolic activity of branched-chain amino acids (BCAAs) (leucine, valine, and isoleucine). Branched-chain α-ketoacids (BCKAs) are produced by branched-chain amino acid aminotransferase (BCAT) starting from BCAAs; then, after being transported into mitochondria, they are decarboxylated by the mitochondrial branched-chain α-ketoacid dehydrogenase (BCKD). The final products (e.g., acetyl-CoA) enter the TCA cycle. By producing acetyl-CoA they are also precursors of FAs. Downregulation of BCAA oxidation enzymes and their relative transcription genes has been found in the AT of patients with insulin resistance and obesity, often associated with elevated levels of circulating BCAAs [72].

The mitochondrial metabolism (mitochondrial energy metabolism, and UCP1 expression/activity) in BAT tissues is also impaired by obesity, as reported in clinical and preclinical studies [73,74].

A preclinical study by Li et al. indicated that brown metabolic features such as thermogenic gene expression, oxygen consumption rate, and lipolysis were compromised in preadipocytes isolated from SAT biopsies of obese individuals [75].

Fatty acids (FA) are important substrates for mitochondrial BAT metabolism. FA uptake was higher in cold-stimulated BAT lean subjects than in obese subjects [76].

A better understanding of the biochemical mechanisms underlying mitochondrial metabolism in obesity could be crucial for the management of obesity.

## 5. Connections between Gut Microbiota and AT Mitochondria in Obesity

### 5.1. Gut Microbiota

Over the past years, a plethora of studies have pinpointed the role of microbiome–host interactions in human health and diseases, as a result of the rapid improvement and availability of next generation sequencing-based techniques. The human intestinal microbiota is a complex and dynamic ecosystem, composed of archaea, protozoa, fungi, viruses and bacteria, the latter being one of the most present and studied fields in recent years.

A different concentration gradient of microbes is observed along the gastrointestinal tract; the upper intestine presents an accumulation of 10–10^3^ cells/g, whereas in the colon there is a much higher number of bacteria ranging from 10^11^ to 10^12^ cells/g, about 0.2 kg of weight in a healthy adult man of 70 kg [77,78]. Exceptional symbiotic interactions have evolved between humans and microbes, and this balance is essential for human health. Gut microbes play key roles in several host physiological processes, such as maintaining intestinal barrier integrity [79], protecting against pathogens [80], influencing the immune system, [81] and metabolism [82], in addition to having profound effects on the gut–brain axis [83,84].

The gut microbial ecosystem starts developing from birth, or even before in the uterus [85], and can be affected by different factors such as mode of delivery, infant feeding, lifestyle, host genetics, drug consumption, and diet [86,87]. Furthermore, studies on age-dependent gut microbiota changes show a clear increase from birth to adulthood which subsequently decreases in old age [88]. Interestingly, in centenarians, a richer and more diverse gut microbiome has been observed as compared to young individuals [89], emphasizing the crucial role of gut microbiota diversity in favouring healthy aging. Furthermore, a recent review highlighted that in addition to the greater bacterial diversity, the presence of some taxa such as *Akkermansia* and *Christensenellaceae* may promote gut homeostasis and healthy aging by reducing adiposity, inflammation, and the consequent risk of developing metabolic and cognitive dysfunction [90].

Many studies have focused on defining what is a “normal” gut microbiota and their mutualistic role in promoting a healthy state (eubiosis). Early studies based on fecal 16S rRNA gene sequencing have identified the bacterial community of the distal large intestine, demonstrating that healthy subjects are characterized by high taxonomic diversity, high microbial genetic richness, and stable core microbiome composition [91]. However, these studies have also shown that even healthy individuals differ markedly in their bacterial composition, highlighting the complex relationship between environment, lifestyle, diet, host genetics, and early microbial exposure [92]. At taxonomic level, the most abundant phyla in the human gut are Bacillota (ex-Firmicutes) and Bacteroidota (ex-Bacteroidetes), representing approximately 90% of gut microbiota, whereas Actinomycetota (ex-Actinobacteria), Pseudomonadota (ex-Proteobacteria), Fusobacteria, and Verrumicrobia are less present [93]. It should be noted that even if some bacteria are less present, this does not necessarily indicate a minor role at a functional level.

In addition to the importance of bacterial composition, it has rapidly become evident that microbial metabolites also play a crucial role. All the substrates necessary to maintain biological functions are supplied through diet, and some of these can only be metabolized by the intestinal microbiota.

The short-chain fatty acids (SCFAs), a group of carboxylic acids known to be involved in the regulation of glucose, lipid metabolism, and inflammation, are produced by the microbial fermentation of dietary fibres and resistant starch. The most abundant and best characterized SCFAs are acetate (C2), propionate (C3), and butyrate (C4) [94], which are mainly produced and absorbed in the cecum and colon (~95%); only a small part (~5%) is excreted in the faeces [95,96]. The molar ratio of acetate:propionate:butyrate in the intestinal lumen is ~57:22:21, whereas a ratio of 71:21:8 has been observed in portal blood. SCFAs can be passively absorbed but most of them are transported through volume-regulated anion channels driven by Na^+^ efflux [97,98]. Notably, butyrate is the primary energy source of colonocytes [99].

SCFAs further act as ligands for G protein-coupled receptors (GPRs) GPR109A and GPR43 and GPR41, which have recently renamed free fatty acid receptors 2 and 3 (FFAR-2 and FFAR-3). When recognized by FFAR-3 and FFAR-2 localized at the surface of the enteroendocrine L cells, they induce the release of satiety hormones, glucagon-like peptide-1 (GLP-1), and peptide YY (PYY) [100,101,102], regulating the host’s food intake. Interestingly, these receptors are not only expressed in the gut, but can also be highly expressed in other tissues, such as AT, liver, muscles, pancreas, and immune cells [82]. A portion of SCFAs can be transported by the portal system to the liver and used in the gluconeogenesis and lipogenesis processes. Finally, SCFAs reach the bloodstream, where they directly modulate the host’s metabolism (see below). The metagenomic characterization of the main SCFA-producing bacteria and the SCFA responsive pathways have been extensively described elsewhere [103,104]. Currently, *Ruminococcus* spp., *Prevotella* spp., *Bifidobacterium* spp., *Lactobacillus* spp., *Bacteroides* spp., *Bacteroides vulgatus*, and *Akkermansia muciniphila* are known to be the main producers of acetate and propionate [105,106], whereas *Coprococcus* spp., *Eubacterium* spp., *Roseburia* spp., and *Faecalibacterium prausnitzii* are mainly butyrate-producing bacteria [107]. These classifications are constantly being reviewed, because metabolic interconnections between different types of bacteria have been found, i.e., acetate produced by *Bacteroidota* species can be utilized by species of *Bacilota* to produce butyrate [108].

A diet rich in proteins of animal origin can lead to a decrease in SCFAs and an increase in BCAAs [109]. Almost half a century ago, it was shown that high levels of BCAAs in the bloodstream were associated with obesity and T2D [110]. Following this, several studies carried out both in animal models and in humans indicated a causal role of BCAAs in obesity, insulin resistance, and the subsequent development of type 2 diabetes. The high levels of circulating BCAAs in obese patients is probably due to the impairment of their catabolic pathways in liver and SAT [111]. The BCAAs are essential amino acids mainly produced and degraded by the intestinal microbial community, and *Clostridia* and *Peptostreptococci* are the main bacterial species involved in their fermentation [112]. However, the scarcity of studies and the functional redundancy of microbial genomes make the identification of the bacterial taxa responsible for BCAA production and their host metabolic impact problematic [113].

### 5.2. Gut Microbiota Changes in Obesity

The first evidence of a link between obesity and gut microbiota derives from studies in germ-free (i.e., microbiota depleted, GF) mice, which under a high-fat diet regimen are better protected against obesity versus their counterpart with microbiota. As further proof, GF mice undergoing faecal microbiota transplantation (FMT) from obese animals showed significant weight gain compared to FMT from lean animals [114]. More recently, the association between gut microbiota and obesity was also observed in humans. Several lines of investigation have revealed that people with obesity are characterized by an imbalance in gut microbial composition and function (dysbiosis) when compared to healthy normal-weight subjects [115]. Furthermore, low faecal bacteria diversity resulted as being associated with total adiposity, dyslipidemia, impaired glucose metabolism, and low-grade inflammation [116]. The first studies in animal models of obesity and in humans have shown that gut microbiota of obese subjects was characterized by a higher abundance of Bacillota and a reduction in the amount of Bacteroidota in comparison to normal-weight subjects [114]. Although further studies replicated a similar trend, a few studies have observed no changes or even reported a reduction in the Bacillota/Bacteroidota ratio [117,118,119,120,121,122,123,124]. It is thus now clear that this simple taxonomic characterization is not sufficient to fully understand the impact of microbiota on the metabolic health status of the host: endogenous and exogenous host factors and bacterial interconnections and functions must also be taken into consideration [125].

In an elegant comparative study, the gut microbiota were analyzed in metabolically healthy obese (MHO, 317) versus metabolically unhealthy obese (MUO, 430) individuals. A significant reduction in gut microbiota diversity was observed in obese patients affected by several comorbidities versus their MHO counterpart. The taxonomic analysis revealed an overall significant abundance of SCFA-producing bacteria such as *Oscillospira* and *Clostridium* genus in the MHO versus MUO subjects [126].

Clear evidence of a direct involvement of the gut microbiota in controlling obesity was provided by the important changes in microbiota composition associated with weight and mass fat loss observed in obese patients after bariatric surgery. GF mice colonized with stools obtained from bariatric patients showed a reduced fat mass gain versus their counterpart colonized with pre-surgical faeces [127]. Bariatric surgery, besides significantly reducing body weight and fat mass, ameliorates or even leads to the remission of T2D and metabolic syndrome [116,128].

Notably, in a very recent study, the plasma levels of SCFAs in patients with severe obesity undergoing bariatric surgery have been investigated both 6 and 12 months after intervention. In association to body weight loss, significant changes in the total SCFAs were found one year after surgery. Interestingly, a negative correlation between BMI and propionate, butyrate, and isobutyrate was also revealed. Conversely, the circulating levels of acetate, valerate, hexanoate, and heptanoate decrease after bariatric surgery. In particular, the authors underline that the increased levels of isobutyrate are significantly correlated with the HOMA-IR, leading to the recovery of insulin sensitivity [129].

### 5.3. Molecular Mechanisms of Gut Microbiota and AT Mitochondria Crosstalk in Obesity

The crosstalk between gut microbiota and mitochondria has been mainly characterized in colonocytes, liver, pancreas, skeletal muscle, and BAT, but less is known about how gut microbiota is mediated by mitochondrial functionality in WAT, especially in the context of obesity in humans [130,131,132].

As described above, many studies have shown that obesity is characterized by mitochondrial dysfunction, which in mature adipocytes is associated to the dysregulation of key metabolic pathways [39,40,44,59,62].

Gut microbiota is now considered as an organ able to modulate, depending on its quality and diversity, the host’s metabolic balance, including mitochondrial function [133]. Several years ago, the involvement of gut microbiota in host metabolism regulation (i.e., body fat mass, glucose tolerance, and insulin resistance) was extensively demonstrated in GF mice colonized with gut microbiota isolated from obese patients [60,134]. Moreover, it was shown that microbiota-depleted mice showed not only an increase in the expression of fat browning markers (UCP1, PPARγ, PGC-1α, and Cidea) but also an improvement of insulin sensitivity and glucose tolerance [60,134].

**Figure 3:**
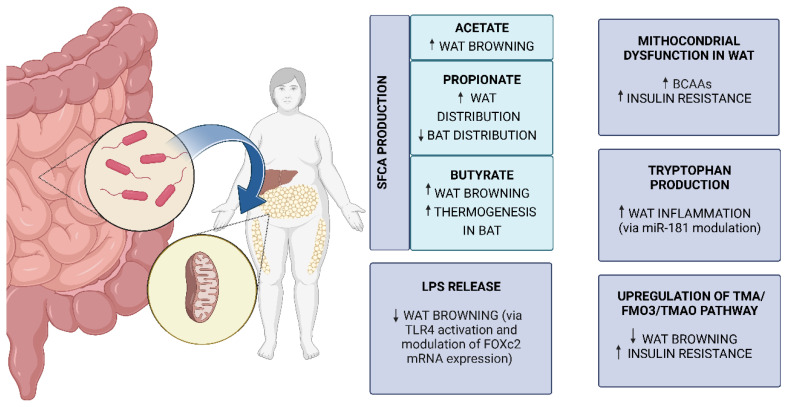
Gut microbiota and microbial metabolites’ influence on WAT mitochondria in obesity. The interplay between gut microbiota and WAT mitochondria is mediated by microbial metabolites such as SCFAs, LPS, BCAAs, tryptophan, and trimethylamine. Among these, SCFAs are the most studied. Interestingly, modulating WAT browning is one of the most frequently reported effects of many microbial metabolites on WAT mitochondria. Created with BioRender.com (accessed on 13 March 2023). Abbreviations: BAT, brown adipose tissue; FMO3, flavin-containing monooxygenase 3; FOXc2, Forkhead box C2; miR-181, micro-RNA 181; TLR4, Toll-like receptor 4; TMA, trimethylamine; TMAO, trimethylamine-N-oxide; WAT, white adipose tissue.

As we will describe below, gut microbiota is able to regulate mitochondrial function in WAT, thus promoting the browning process and subsequently improving energy expenditure and metabolism (Figure 3).

Several lines of evidence suggest that SCFAs play a crucial role in host metabolism regulation, although contradictory results have been obtained [134]. Indeed, SCFAs are more present in faeces of subjects with obesity compared to lean individuals and of ob/ob mice, suggesting that their concentration is correlated to obesity. On the other hand, oral administration of SCFAs both in humans and in mice has been found to have positive effects on weight loss and adiposity [134]. Recently, it has been shown that once SCFAs have entered the systemic circulation, they can activate proteins involved in regulating mitochondrial function, such as the uncoupling protein 2 (UCP 2), the 5′ AMP-activated protein kinase (AMPK) and the acetyl-CoA carboxylase (ACC), thus leading to down-regulation of the peroxisome proliferator activated receptor gamma (PPAR-γ) and to activating mitochondrial OXPHOS [135]. More specifically, SCFAs can induce the browning process and triglyceride hydrolysis [133,134]. The role of SCFAs in preventing obesity was demonstrated several years ago by in vivo studies which emphasized the role of FFAR2 in suppressing insulin signalling in adipocytes, thus halting the accumulation of lipids in AT [136]. FFAR2 has also been reported to modulate mitochondrial biogenesis in brown adipocytes [137] and it is now well known that FFAR2 can inhibit the circulating lipoprotein lipase which promotes the uptake and storage of fatty acids in adipocytes [138].

Amongst the SCFAs, butyrate seems to play an important regulatory role in metabolism by modulating energy homeostasis in BAT and WAT, as well as in liver. Studies conducted by supplementing butyrate have shown UCP1-induced thermogenesis in BAT and browning of WAT mediated by the increase in PGC-1α, a master regulator of mitochondrial biogenesis [139,140].

With regard to propionate, contrasting results have been obtained. Some studies have suggested that its levels in serum are positively correlated to obesity in humans and that it causes weight gain and insulin resistance in mice [141]. In particular, it has been reported that a protein of the inner membrane of mitochondria in BAT, acyl-CoA synthetase short-chain family member 3 (ACSS3), is crucial to propionate metabolism. Defects in ACSS3 lead to propionate accumulating in serum with a consequent decrease in BAT, increase in WAT, and insulin resistance [142]. On the contrary, propionate has been reported to inhibit lipolysis and to promote adipose tissue lipid buffering, leading to a positive impact on fat accumulation and insulin sensitivity by modulating FFAR2 expression [134].

Additionally, in the case of acetate (the most abundant circulating SCFA), contrasting results have been obtained, probably due to different methods of administration. In any case, in vitro and animal studies suggest that acetate is an inducer of WAT browning [143].

It has become clear that the gut can be colonized by pathobionts with negative effects on both host metabolism and inflammation. For instance, three pathobionts (*Oscillibactervalericigenes*, *Barnesiellaviscericola*, and *H. saccharovorans*) have been shown to damage mitochondrial OXPHOS in WAT, thus promoting insulin resistance both in mice and humans [144]. Several lines of evidence show that the negative effects on energy metabolism and WAT inflammation are linked to the lipopolysaccharide (LPS) signalling pathway [145]. LPS is a component of the bacterial membrane which enters the bloodstream through gut permeability and is able to activate an immune and inflammatory response [133]. In particular, it has been shown to activate Toll-like receptor 4 (TLR4), consequently inhibiting WAT browning and inducing mitochondrial dysfunction [145]. Moreover, LPS can inhibit WAT browning through modulating mRNA expression of the Forkhead box C2 (Foxc2), a transcription factor which regulates expression of browning factors, such as UCP1, PGC-1α, and PR domain-containing 16 (Prdm16) [145].

In addition to SCFAs, several other metabolites produced by microbiota seem to be involved in metabolic processes and in obesity [146]. Among these, products derived from the metabolism of tryptophan have been shown to be crucial to tuning host metabolism, adipogenesis, and WAT inflammation by modulating a micro-RNA (miRNA), miR-181. Interestingly, dysbiosis induced in mice by a high-fat diet has been linked to the over-expression of miR-181 in WAT adipocytes and to insulin resistance, obesity, and WAT inflammation. Even in this case, the molecular pathway involved in energy expenditure seems to depend on UCP1-mediated thermogenesis in epididymal WAT, but not in inguinal WAT or BAT [147].

Another mechanism of modulating WAT browning by gut microbiota metabolites, closely linked to obesity and its comorbidities, is the trimethylamine/flavin-containing monooxygenase-3/trimethylamine-N-oxide (TMA/FMO3/TMAO) pathway. This pathway is considered an endocrine axis which joins gut microbiota and host. It has been shown that a typical Western diet—which is particularly enriched in L-carnitine, phosphatidyl-choline, and choline—generates TMA, a substrate for gut microbiota. TMA is converted to TMAO, whose high levels in blood have been linked to T2D and cardiovascular disease in humans, by the hepatic enzyme FMO3. In vivo studies have shown that FMO3 knockdown is linked to the upregulation of WAT browning players, such as β1-adrenergic receptor (Adrb1) and UCP1 [146].

The importance of gut microbiota metabolites in modulating WAT mitochondrial function has also emerged from studies on intermittent fasting, shown to increase the Bacillota:Bacteroidota ration, and thus the production of acetate and lactate which induce browning [145,146].

It should be noted that the crosstalk between gut microbiota, mitochondria, and WAT has been mainly analysed by in vitro and animal studies. In humans, a study on the association between gut microbiota composition and browning markers in subcutaneous and visceral fat has been conducted [148]. The results highlighted that subjects with severe obesity and insulin resistance were characterized by a decrease in the abundance of Bacillota and, in particular, of the *Ruminococcaceae* family, when compared to obese subjects with normal insulin sensitivity. This decrease was also correlated with an mRNA decrease in browning markers in SAT, but not in VAT. Although the mechanism behind this correlation is still unknown, one intriguing hypothesis is that acetate, the main metabolite produced by Bacillota, may be responsible for insulin sensitivity and AT browning, in agreement with previous studies [145]. It should also be emphasized that studying browning in humans is hampered by the low expression of brown and beige markers.

## 6. Conclusions

AT dysregulation has been recognized as a key process in the pathophysiology of obesity and its cardiometabolic complications. In the last few years, there has been growing interest in investigating the underlying molecular basis of adiposopathy. In this context, mitochondria, organelles that adapt their biological response to the inner and outer environment, have emerged as crucial regulators of both WAT and BAT metabolism and function, thus acquiring the potential to becoming keystones in the treatment of obesity and its comorbidities. Given that gut microbiota and microbial metabolites have been increasingly recognized as capable of tuning host metabolism, adipogenesis, and WAT inflammation, we hypothesized that their dysregulation in obesity could affect AT metabolism by exerting direct and indirect effects on AT mitochondria.

Through collecting and revising the current evidence on connections between gut microbiota and AT mitochondria in obesity, we have gained insight into the molecular biology of their hitherto largely unexplored crosstalk, tracing how gut microbiota and its metabolites may regulate AT mitochondrial function, mainly through the modulation of WAT browning and, consequently, host energy expenditure and metabolism. Further studies are needed to translate these fundamental findings into clinical practice in terms of both diagnostic and therapeutic opportunities for individuals affected by obesity and its cardiometabolic complications.

## Figures and Tables

**Table 1:** WAT and BAT mitochondria.

Mitochondrial Characteristics	WAT	BAT
**Content**	Lower	Higher
Dimension *	Smaller	Bigger
Shape *	Elongated	Spherical
Inner membrane cristae *	Less packed	More packed
**UCP-1 expression**	Lower	Higher
**Main function**	Energy storage and lipid homeostasis	Non-shivering thermogenesis

Abbreviations: BAT, brown adipose tissue; UCP-1, uncoupling protein-1; WAT, white adipose tissue. * studies on rats.

## Data Availability

Not applicable.
